# Associations of Transforming Growth Factor-β (TGF-β) with Chronic Kidney Disease Progression in Patients Attending a Tertiary Hospital in Johannesburg, South Africa

**DOI:** 10.3390/biomedicines14010236

**Published:** 2026-01-21

**Authors:** Alfred Meremo, Raquel Duarte, Caroline Dickens, Therese Dix-Peek, Deogratius Bintabara, Graham Paget, Saraladevi Naicker

**Affiliations:** 1 Department of Internal Medicine, School of Clinical Medicine, Faculty of Health Science, University of the Witwatersrand, Johannesburg 2193, South Africa; raquel.duarte@wits.ac.za (R.D.); caroline.dickens@wits.ac.za (C.D.); therese.dix-peek@wits.ac.za (T.D.-P.); graham.paget@wits.ac.za (G.P.); saraladevi.naicker@wits.ac.za (S.N.); 2 Department of Internal Medicine, School of Medicine & Dentistry, The University Dodoma, Dodoma 11000, Tanzania; 3 Department of Community Medicine, School of Medicine & Dentistry, The University Dodoma, Dodoma 11000, Tanzania; deogratius.bintabara@udom.ac.tz

**Keywords:** transforming growth factor-beta (TGF-β) isoforms, prediction, CKD progression, Johannesburg, South Africa

## Abstract

**Introduction:** The global prevalence of chronic kidney disease (CKD) is increasing and it is associated with higher mortality rates. Transforming growth factor-beta (TGF-β) can serve as a novel biomarker for early prediction of chronic kidney disease (CKD) progression. **Methods:** This was a prospective longitudinal study among black patients with CKD who attended the Charlotte Maxeke Johannesburg Academic Hospital between September 2019 and March 2022. Patients provided urine and blood samples for laboratory investigations at study entry (0) and at 24 months follow up. Baseline serum and urine TGF-β1, TGF-β2 and TGF-β3 levels were measured using ELISAs. Multivariable logistic regression analysis was utilized to determine if TGF-β isoforms could predict CKD progression. **Results:** A total of 312 patients were enrolled at baseline, of whom 275 (88.1%) had early-stage CKD (Stage 1–3). A majority, 95.2% (297/312), of the patients completed the study after 2 years follow up. The prevalence of CKD progression was 47.8% when measured by a sustained decline in eGFR of >4 mL/min/1.73 m^2^/year or more and 51.9% when measured by a change in uPCR > 30%. The patients with CKD progression had significantly lower eGFR and increased urine protein–creatinine ratios compared to non-progressors. Furthermore, comparing progressors with non-progressors, the median serum TGF-β1 was 21210 (15915–25745) ng/L vs. 24200 (17570–29560) ng/L and the median urine TGF-β3 was 17.5 (5.4–76.2) ng/L vs. 2.8 (1.8–15.3) ng/L, respectively. Baseline serum and urine TGF-β isoforms were unable to discriminate between CKD progressors and non-progressors after multivariable logistic regression analysis. **Conclusions:** Despite the multiple roles of TGF-β isoforms in kidney disease, baseline levels were not predictive of chronic kidney disease progression.

## 1. Introduction

The global prevalence of chronic kidney disease (CKD) is approximately 10% and is associated with higher mortality rates in low- and middle-income countries in sub-Saharan Africa, including South Africa, which are the most disadvantaged countries, with inadequate resources and preparedness to provide kidney replacement therapies [1,2]. The prevalence of CKD in sub-Saharan Africa is approximately 18%, and it is 11% in South Africa [3,4]. This burden of chronic kidney disease (CKD) in sub-Saharan Africa presents more frequently in the elderly and women, and CKD progression is accelerated by hypertension, diabetes mellitus and obesity [4,5]. Chronic kidney disease is a common disorder associated with significant health care expenses, resulting in cardiovascular events, end-stage kidney disease (ESKD) and eventually death [6,7]. Chronic kidney disease can silently advance to ESKD, thus earlier detection is important for instituting timely preventative measures [8,9].

Albuminuria, serum creatinine and cystatin-C are key and frequently utilized predictive models for CKD progression; however, these routinely used biomarkers show changes relatively late in the disease processes [10,11]. Multi-omic approaches which integrate genomics, proteomics, metabolomics and transcriptomics are reshaping chronic kidney disease classification, diagnostic accuracy, treatment and prognosis [12,13]. Studies have reported the expression of a number of novel biomarkers, including transforming growth factor-β (TGF-β), microRNAs (miRNAs), tumor necrosis factor-α (TNF-α), matrix metalloproteinases (MMPs), uromodulin, IL-18, liver-type fatty acid-binding protein (L-FABP), monocyte chemoattractant protein 1 (MCP-1), kidney injury molecule-1 (KIM-1) and α-1 microglobulin, amongst others, which may precede alterations in albuminuria, serum creatinine and cystatin-C [10,14,15]. Despite these innovations, challenges remain as to the roles of novel biomarkers and their integration into clinical practice, potentially leading to early intervention and improved patient outcomes [13,16].

Transforming growth factor-β commonly modulates kidney fibrosis and inflammation and has contrasting roles in kidney fibrosis and inflammation via Smad-dependent mechanisms requiring Smad2, Smad3, Smad4 and Smad7 and specifically through Smad3-dependent non-coding RNAs [17,18]. Smad2 is protective while Smad3 is pathogenic which results in kidney inflammation and fibrosis culminating in CKD and ESRD [19,20]. Transforming growth factor β1 (TGF-β1) is regarded as the principal profibrotic mediator in most kidney diseases and has been the most extensively studied isoform to date [21,22]. Laboratory studies have indicated that TGF-β1 has some value in diagnosing glomerular diseases and could be used as a biomarker for predicting recovery of glomerular diseases and CKD progression [23,24]. Patients with CKD and human immunodeficiency virus (HIV) showed higher urine and serum TGF-β1 levels, especially in early CKD stages [25,26].

A study of people living with HIV (PLWH) showed that urinary TGF-β1 and TGF-β2 levels were increased in PLWH who had CKD [27]. The intensity and persistence of TGF-β2 influence CKD progression affects cellular apoptosis and tubular degeneration by promoting fibrosis. TGF-β2 is involved in the inflammatory response and oxidative stress, which exacerbates renal damage [28]. TGF-β3 has a protective role in immune-mediated responses [29,30]. The roles of TGF-β2 and TGF-β3 in CKD are less clear and there is a paucity of data on TGF-β1, TGF-β2 and TGF-β3 isoforms as biomarkers of CKD progression. The aim of this study was to evaluate whether baseline serum and urinary TGF-β isoforms could predict CKD progression among patients attending a tertiary hospital in Johannesburg, South Africa.

## 2. Methods

### 2.1. Study Design, Population and Settings

A prospective longitudinal study to investigate novel biomarkers that predict CKD progression among black patients attending the Charlotte Maxeke Johannesburg Academic Hospital was carried out between September 2019 and March 2022. The enrolment period was from September 2019 to March 2020 and follow up was conducted at 24 months, from September 2021 to March 2022. Inclusion criteria for the study were patients who were >18 years of age and able to give informed consent. Patients with CKD stages 1–4 who had controlled hypertension (blood pressure < 140/90 mm Hg) and diabetes mellitus (HbA1c < 7%) and who had been attending the kidney outpatient clinic (KOPD) for at least 6 months were recruited. Patients with active malignancies, active infections, autoimmune diseases and who did not self-report black ethnicity were excluded. Black ethnicity is associated with faster and significantly increased rates of CKD progression and ESKD and thus comprised the study population [31,32].

### 2.2. Data Collection and Laboratory Procedures

Study was conducted between September 2019 and March 2022. The enrolment period was from September 2019 to March 2020 and follow up was conducted at 24 months, from September 2021 to March 2022. The sample size was obtained using the Krejcie and Morgan formula (1970) for prospective studies [33]. The minimum sample size calculation was based on a study conducted in northern California on adults with CKD [34]. Therefore, the minimum required sample size of this study was 300 patients who had CKD in order to have stratification with at least 250 patients in Stages 1–3 and 50 patients in CKD stage 4. A consecutive sampling technique was used whereby every patient who met the inclusion criteria during the study period was enrolled into the study until the necessary sample size was reached [35]. Demographic and clinical data comprising gender, age, weight, height, BMI, etiology of CKD, glycaemic status, history of smoking and medications were retrieved from the KOPD clinic data; face-to-face interviews at enrolment included completion of a questionnaire. Vital signs were measured at recruitment and at follow up.

Systolic and diastolic blood pressure was assessed three times at a clinic visit and the mean of the second and third readings were used. Body mass index (BMI) was determined using the National Health Services (NHS-UK) BMI calculator [36]. Patients provided blood and urine samples at enrolment and at 24 months follow up for routine laboratory investigations; the samples were assayed by the National Health Laboratory Services (NHLS) utilizing the Cobas 6000 analyzer (Roche Diagnostics, Indianapolis, IN, USA). Measurements of serum creatinine, serum transferrin levels, urinary protein creatinine ratio (uPCR), electrolytes, full blood picture (FBP), random blood glucose (RBG)/fasting blood glucose (FBG), glycosylated hemoglobin (HbA1C), high density lipids (HDL) cholesterol, calcium and phosphate, as required by the standard of care, were recorded at the time of the first visit and at 24 months follow up.

Anemia was defined as a hemoglobin (Hb) concentration of <12.0g/dL in females and <13.0 g/dL in males [37]. The isotope dilution mass spectrometry (IDMS) traceable enzymatic assay was utilized to calculate serum creatinine and the Chronic Kidney Disease Epidemiology Collaboration (CKD-EPI) equation (without using the African American correction factor) was utilized in estimating the glomerular filtration rate (eGFR) [38].

Chronic kidney disease progression was assessed using the four most common definitions: (i) continued decrease in eGFR of >4 mL/min/1.73 m^2^/year or more [34]; (ii) change to a more severe stage of CKD [39]; (iii) more than 30% reduction in eGFR in two years, whereby percent change in eGFR was calculated as (follow up eGFR–baseline eGFR)/baseline eGFR × 100 [40]; and (iv) more than 30% change in uPCR in two years, whereby change in uPCR was calculated as (follow up uPCR–baseline uPCR)/baseline uPCR × 100 [41].

### 2.3. Measurement of Serum and Urine TGF-β Isoforms

In addition to samples for routine clinical measurements, baseline serum and urine samples were collected for biomarker quantification. Serum samples were stored at −80 °C while urine samples were centrifuged at 4000× *g* rpm for 20 min and the supernatant stored at −80 °C. Human TGF-β1, TGF-β2 and TGF-β3 DuoSet ELISAs (R&D Systems, Inc., Minneapolis, MN, USA) were utilized to determine the baseline serum and urine TGF-β1, TGF-β2 and TGF-β3 levels, in accordance with the manufacturer’s instructions. To 50 µL of serum or urine, 5 µL of 1 N HCl was added to activate the latent TGF-βs and a 1.2 N sodium hydroxide/0.5 M HEPES solution was used for neutralization, resulting in a 1 in 2 sample dilution. Serum samples assayed for TGF-β1 were further diluted to a final dilution of 1 in 100. A microplate reader (Biotek 800TS, Agilent, Santa Clara, CA, USA) was set to 450 nm with background correction set at 650 nm to determine optical densities. The standards were fitted to a 5-parameter logistic curve (www.myassays.com, accessed on 6 April 2023) to determine biomarker concentrations within the samples at baseline.

### 2.4. Data Analysis

*REDCap* (Research Electronic Data Capture), hosted at Vanderbilt University, was utilized for data entry and processing [42,43], and data analysis was performed utilizing STATA version 17 (College Station, TX, USA). To summarize demographic and clinical data, descriptive statistics were utilized. Data were checked for normal distribution by making use of the Shapiro–Wilk test, which showed that all continuous variables did not meet the normality assumption, thus medians with their interquartile ranges were employed as the measure of the center and dispersion, respectively, to summarize those continuous variables. Furthermore, for the comparison between continuous variables and outcome variables, the Wilcoxon rank-sum test was employed. Discrete variables were recorded as frequencies and proportions; to test for associations between variables and CKD progression, Pearson’s chi-square test was utilized. To determine the effect of the variables at baseline and changes in the variables on CKD progression at 24 months, multivariable logistic regression analysis was utilized. Variables with a *p*-value of less than 0.2 on univariable logistic regression models were then entered into the multivariable logistic regression models with the addition of age as an adjusting variable; variables with a *p*-value of less than 0.05 were thought to have significant power of association. The associations of urinary and serum TGF-β isomers with CKD progression were assessed by odds ratios. This analysis was repeated with the four definitions of CKD progression as independent variables. A *p*-value of less than 0.05 was considered significant.

## 3. Results

### 3.1. Study Participants

**Figure 1:**
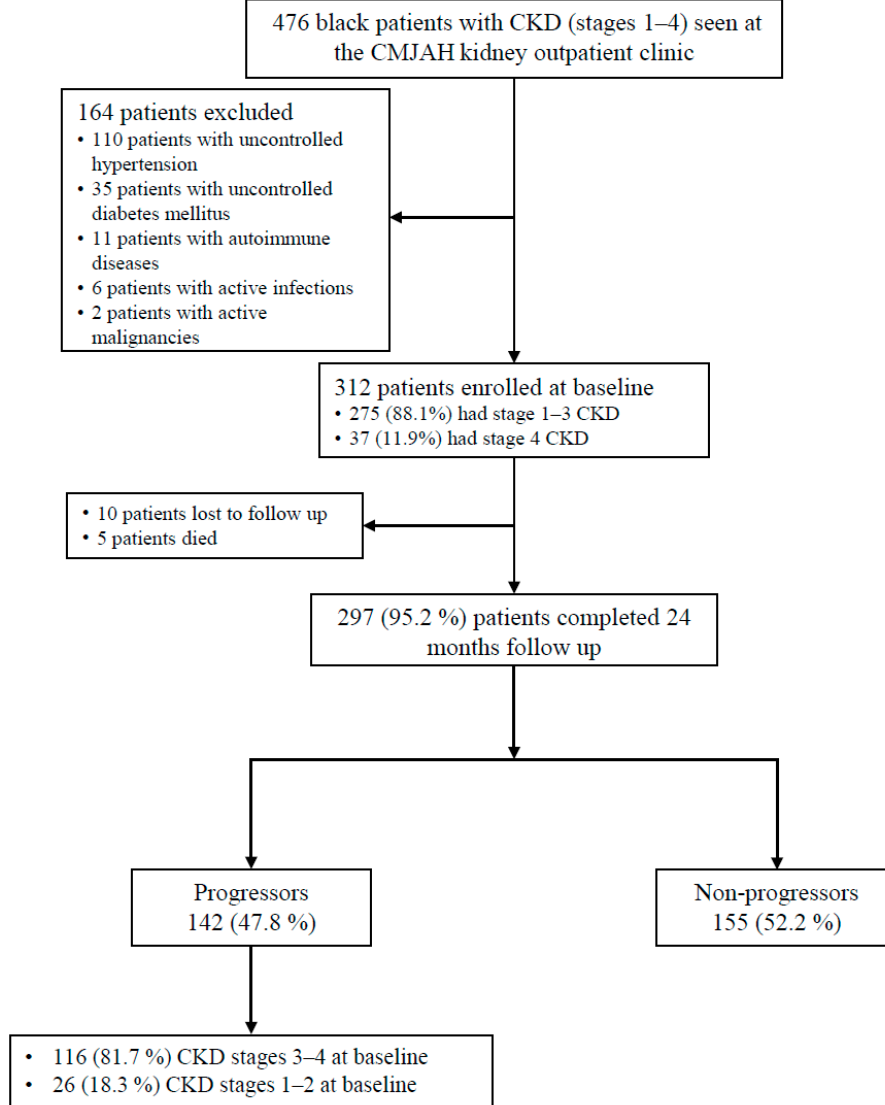
Enrollment of CKD study participants. Progression is based on a sustained decline in eGFR of >4 mL/min/1.73 m^2^/year or more. CKD, chronic kidney disease; CMJH, Charlotte Maxeke Johannesburg Academic Hospital.

During the study period, of the 476 patients visiting the KOPD clinic for their routine follow up, 312 patients were enrolled at baseline, of whom 275 (88.1%) had early-stage CKD (CKD stages 1–3). The majority (*n* = 297, 95.2%) of the patients completed the study; by the 2-year follow up, CKD progression had occurred in 142 (47.8%) of the patients, of whom 116 (81.7%) had stage 3 or 4 CKD at baseline (Figure 1).

### 3.2. Prevalence of CKD Progression Using Clinical Biomarkers

**Figure 2:**
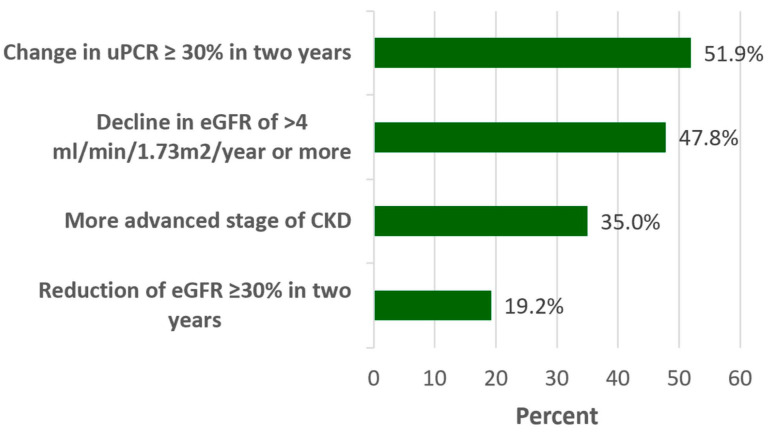
CKD progression after 2 years based on clinical biomarkers (eGFR and uPCR). Upcr, urinary protein creatinine ratio; eGFR, estimating the glomerular filtration rate; CKD, chronic kidney disease.

At 24 months follow up, CKD progression occurred in 142 (47.8%) patients who had a sustained decrease in eGFR of >4 mL/min/1.73 m^2^/year or more; 104 (35%) patients moved to a more severe stage of CKD; 57 (19.2%) patients had >30% reduction in eGFR in two years and 154 (51.9%) patients had a change in uPCR > 30% in two years (Figure 2). Unless otherwise stated, subsequent analysis used the continued decrease in eGFR of >4 mL/min/1.73 m^2^/year or more model (Figure 2).

### 3.3. Demographic and Clinical Characteristics of CKD Progressors and Non-Progressors

There was no significant difference between progressors and non-progressors based on sex, age, marital status, education or employment (Table 1). The significant variables were lower baseline median eGFR and increased median uPCR in CKD progressors than in non-progressors. Baseline serum calcium and Hb were lower in CKD progressors than in non-progressors, while baseline serum phosphate was higher in progressors versus non-progressors (Table 1).

Baseline serum TGF-β1 was lower in CKD progressors vs. non-progressors, as shown by the median (IQR) (21,210 (15,915–25,745) ng/L vs. 24,200 (17,570–29,560) ng/L; *p* = 0.004) using the sustained eGFR decline model. Similar results were seen for the models exploring changes in CKD stage and percent reduction in eGFR, but there was no significant change in TGF-β1 levels when using the 30% increase in uPCR model. Baseline median (IQR) urine TGF-β3 was 17.5 (5.4–76.2) ng/L for those with CKD progression and 2.8 (1.8–15.3) ng/L for those without CKD progression using the sustained eGFR decline model (*p* = 0.017) but no significant differences were seen for this marker in any of the other models (Table 2). Median serum TGF-β2 levels did not differ significantly between progressors and non-progressors regardless of the model used.

Sensitivity analysis was repeated for those recruited with early-stage CKD (stages 1 and 2) and again with those recruited with late-stage CKD (stage 3a, 3b and 4). For those patients recruited with CKD stages 1 and 2, 40% (26/65) showed CKD progression based on the sustained decline in eGFR model and 62% (40/65) showed progression using any of the four models. In comparison, of those recruited with CKD stages 3 and 4, 50% (116/232) showed CKD progression with the sustained eGFR decline model and 76% (177/232) showed progression with any of the four models.

Patients with CKD stages 1 and 2 who progressed had significantly lower platelet counts and higher uric acid and higher bicarbonate levels than non-progressors (Supplementary Table S1) but showed no significant changes in any of the TGF-β isoforms measured (Supplementary Table S2). Those recruited with CKD stages 3 and 4 mimicked the clinical picture seen in the overall cohort with significant differences in creatinine, eGFR, uPCR, hemoglobin, calcium and phosphate (Supplementary Table S3). Of interest, however, is the calcium phosphate product, which was significantly higher in the progressors than the non-progressors in the late-stage sub-cohort. Medication use in the late-stage sub-cohort mimicked the overall cohort except for calcium channel blockers, which did not show significant differences. The TGF-β profile seen in the CKD stage 3 and 4 cohort reflected the TGF-β profile of the overall cohort, with lower serum TGF-β1 levels in CKD progressors compared with non-progressors for the three models based on eGFR but no difference in the uPCR model. Urine TGF-β3 levels were higher in the sustained decline eGFR model (Supplementary Table S4).

### 3.4. Association of Baseline TGF-β Concentrations with CKD Progression

The previously described four different models for estimating CKD progression showed no changes in the odds of developing CKD progression based on baseline TGF-β isoform levels (Table 3).

## 4. Discussion

Early identification of CKD progression is essential for initiating and improving the management of CKD; albuminuria, serum creatinine and cystatin-C, which form the core predictive models of CKD progression, show alterations when the disease is already advanced. TGF-β has been touted as a candidate biomarker due to its critical role in kidney fibrosis and inflammation [20,44]. TGF-β1 plays a major profibrotic role in most kidney diseases, resulting in inflammation and fibrosis, eventually leading to ESKD [21,22]. Studies have reported that serum and urine TGF-β1 levels were increased in most kidney diseases and were independently associated with lower eGFR and increasing proteinuria [45,46]. We wanted to investigate TGF-βs as potential biomarkers to predict progression of CKD. Given the paucity of information on TGF-β isoforms in kidney disease, this study aimed to shed light on their effects.

Using traditional biomarkers, the prevalence of CKD progression after 2 years of follow up by a sustained decline in eGFR of >4 mL/min/1.73 m^2^/year or more was 47.8%. A similar prevalence of 46.7% was reported in a UK cohort study, while a lower prevalence of 38% was reported from a study in northern California [34,47]. The prevalence of CKD progression denoted by a change in uPCR > 30% was 51.9%, which is similar to a study in Australia where the prevalence of CKD progression measured by change in uPCR was 59%, while a lower prevalence of 26.1% was reported in a study in Japan [48,49]. A possible explanation for the different findings could be related to disparities in different populations and their related complications [50,51].

In this study, both serum and urine TGF-β1 were measured at baseline to assess the utility of TGF-βs as predictors of CKD progression. The majority (97.3%) of our study patients had detectable serum TGF-β1 and 30% of patients had detectable urine TGF-β1 levels. Lower baseline serum and urine TGF-β1 concentrations were associated with CKD progression in the “eGFR-decline”, “eGFR-reduction” and “change to more advanced stage of disease” models. However, baseline serum and urine TGF-β1 did not predict CKD progression in the multivariable analysis. These findings are similar to those from previous studies that have shown a reduction in serum TGF-β1 levels with the decline in eGFR model [52,53]. In spite of the unavailability of clinical studies on the utility of TGF-β1 in predicting CKD progression, laboratory studies have found that serum and urine TGF-β1 were crucial in the progression of glomerular diseases and were significantly associated with CKD progression [24,54]. Amongst the reasons for this could be that the majority of the patients in this study had CKD stages 3–4 at baseline, with lower urinary TGF-β1 levels noted in patients with advanced CKD and patients with controlled diabetes mellitus and hypertension [25,55].

Studies on the role of TGF-β2 in predicting CKD progression are uncommon despite the fact that TGF-β2 is highly expressed in most kidney diseases and are associated with kidney fibrosis [56,57]. In this study, the majority (82.5%) of the patients had detectable serum TGF-β2 and 17.5% patients had detectable urine TGF-β2. Declining eGFR and CKD progression were associated with lower baseline serum TGF-β2 concentrations and increased baseline urine TGF-β2 concentrations. However, baseline serum and urine TGF-β2 did not predict CKD progression. Studies have reported that TGFβ2 induces myofibroblast differentiation, eventually leading to kidney fibrosis, loss of renal function and CKD progression [58,59].

Studies have shown that TGF-β3 induces kidney inflammation and fibrosis, which is less severe when compared to the effects of TGF-β1 [60,61]. In our study, more than half (58.6%) of the patients had detectable serum TGF-β3 and 17.9% patients had detectable urine TGF-β3. CKD progression was associated with declining eGFR and higher baseline urinary TGF-β3. However, baseline urinary TGF-β3 did not predict CKD progression. Previous studies reported that TGF-β3 in the kidney might have antifibrotic and kidney-protective properties unlike the activity of TGF-β1, and that TGF-β3 expression levels peak with advanced CKD stages [60,62]. Our study adds to the pool of evidence that renal TGF-β3 might have roles that oppose or counteract the activity of TGF-β1.

In previous studies examining the utility of urine and serum biomarkers in predicting CKD among patients with HIV/AIDS, urinary TGF-β1 and TGF-β2 concentrations were increased among CKD patients who had HIV infection [25,27]. Studies have reported urinary biomarkers to be more sensitive and specific in predicting CKD progression when compared to serum biomarkers [25,34]. However, in our study, few CKD patients had detectable urine TGF-β at baseline; amongst the reasons for this was that the majority of patients had stage-3–4 CKD, which is known to be associated with lower expression of TGF-β isoforms [25,63]. Also, almost all study patients were hypertensive and were on antihypertensive agents, calcium channel blockers and diuretics, which reduce production of renal TGF-βs [63,64].

Limitations of this study included the inability to detect TGF-β isoforms in the majority of urine samples, limiting the statistical power. Also, the logistic regression models were limited by the relatively few covariates and a modest sample size, as well as the unavailability of uACR in routine patient care data at CMJAH; studies advocate use of both uACR and uPCR [65,66]. A longer follow-up duration of more than 2 years would have been ideal to determine the role of TGF-β isoforms in predicting CKD progression among patients who had controlled hypertension and diabetes mellitus at baseline. Despite these limitations, the results of our study add to the current body of literature on TGF-β isoform expression and CKD progression.

## 5. Conclusions

Patients with CKD progression had lower baseline concentrations of serum TGF-β1 and increased urinary TGF-β3 concentrations compared to non-progressors. However, these isoforms were not predictors of CKD progression. The roles of the various TGF-β isoforms in CKD progression are still unclear and highlight the importance of further studies to determine their isoform specific effects.

## Figures and Tables

**Table 1:** Baseline demographic and clinical characteristics of study patients by CKD progression.

Characteristic	CKD Progression (*n* = 142)	No CKD Progression (*n* = 155)	*p*-Value
	Proportion (%) or Median (IQR)	Proportion (%) or Median (IQR)	
**Demographics:**			
Age (years)	59 (46–67)	56 (45–66)	0.249
Sex Male Female	78 (54.9%) 64 (45.1%)	78 (50.3%) 77 (49.7%)	0.427
Marital status Single Married Widow/Widower Separated/Divorced	38 (26.8%) 77 (54.2%) 18 (12.7%) 9 (6.3%)	51 (32.9%) 77 (49.7%) 19 (12.3%) 8 (5.2%)	0.701
Highest level of education No formal education Primary Secondary Tertiary	18 (12.7%) 30 (21.1%) 51 (35.9%) 43 (30.3%)	16 (10.3%) 37 (23.9%) 44 (28.4%) 58 (37.4%)	0.387
Occupation Unemployed Domestic workers Self employed Public/Private servant Retired	22 (15.5%) 25 (17.6%) 32 (22.5%) 51 (35.9%) 12 (8.5%)	22 (14.2%) 31 (20.0%) 33 (21.3%) 56 (36.1%) 13 (8.4%)	0.985
**Clinical Variables:**			
BMI (kg/m^2^)	30.6 (26.8–35.7)	29.7 (26.0–33.4)	0.268
SBP (mmHg) DBP (mmHg)	140 (132–140) 83 (74–90)	140 (125–140) 82 (73–90)	0.070 0.334
Creatinine (umol/L)	147.5 (118–183)	134 (105–162)	0.004
eGFR (mL/min/1.72 m^2^)	37 (32–51)	44 (34–61)	0.005
uPCR (g/mmol)	0.039 (0.015–0.085)	0.016 (0.008–0.032)	<0.001
FBG (mmol/L)	4.5 (4.2–5.0)	4.4 (4.2–4.9)	0.364
HbA1c (%)	7.0 (6.9–7.0)	7.0 (6.6–7.0)	0.355
Hemoglobin (g/dL)	13.0 (11.7–14.2)	13.7 (12.2–15.3)	0.011
WBC (×10^9^ cells/L)	6.10 (5.07–7.84)	6.47 (4.81–7.74)	0.918
Platelets (×10^9^ cells/L)	251 (211–320)	269 (218–323)	0.475
Uric acid (mmol/L)	0.42 (0.35–0.51)	0.40 (0.31–0.48)	0.020
HDL cholesterol (mmol/L)	1.16 (1.00–1.43)	1.25 (1.01–1.56)	0.153
Calcium (mmol/L)	2.30 (2.23–2.39)	2.34 (2.25–2.42)	0.004
Phosphate (mmol/L)	1.12 (0.93–1.27)	1.02 (0.88–1.16)	0.007
Sodium (mmol/L)	141 (139–143)	141 (138–143)	0.758
Potassium (mmol/L)	4.3 (4.0–4.7)	4.2 (3.8–4.5)	0.054
Bicarbonate (mmol/L)	22 (20–24)	22 (20–24)	0.787
Calcium phosphate product (mmol^2^/L^2^)	2.50 (2.12–2.89)	2.41 (2.03–2.70)	0.092
Medications:			
Diuretics	90 (63.4%)	63 (40.7%)	0.001
ACEIs/ARBs	33 (23.2%)	27 (17.4%)	0.212
Aldactone	6 (4.2%)	5 (3.2%)	0.649
Calcium channel blockers	121 (85.2%)	117 (75.5%)	0.036
Statins	81 (57.0%)	70 (45.2%)	0.041
Oral Hypoglycemic	14 (9.9%)	22 (14.2%)	0.253
Insulin	45 (31.7%)	23 (14.8%)	0.001
Allopurinol	15 (10.6%)	19 (12.3%)	0.647
Junior ASA	41 (28.9%)	28 (18.1%)	0.028
Beta blockers	79 (55.6%)	63 (40.7%)	0.010
Aldomet	35 (24.7%)	34 (21.9%)	0.580
Hydralazine	11 (7.8%)	8 (5.2%)	0.363
Nitrates (ISMN/ISDN)	4 (2.8%)	3 (1.9%)	0.617
Doxazosin	64 (45.1%)	70 (45.2%)	0.987
Others	45 (31.7%)	54 (34.8%)	0.565

CKD, chronic kidney disease; ACEIs, angiotensin converting enzyme inhibitors; ARBs, aldosterone receptor blockers; ASA, acetylsalicylic acid; DBP, diastolic blood pressure; eGFR, estimated glomerular filtration rate; FBG, fasting blood sugar; HbA1c, glycosylated hemoglobin A1C; HDL, high density lipoprotein; IQR, interquartile range; ISMN; isosorbide mononitrate; ISDN, isosorbide dinitrate; uPCR, urine protein creatinine ratio; SBP, systolic blood pressure; WBC, white blood cells; BMI: Body mass index.

**Table 2:** Baseline serum and urine TGF β concentrations of study patients by different CKD progression criteria.

	eGFR Decline > 4 mL/min/1.73 m^2^/year or More			Changed to a More Advanced Stage of CKD			>30% Reduction in eGFR in 2 Years			>30% Increase in uPCR in 2 Years		
Baseline Characteristic	CKD Progression (*n* = 142) Median (IQR)	No CKD Progression (*n* = 155) Median (IQR)	*p*-Value	CKD Progression (*n* = 104) Median (IQR)	No CKD Progression (*n* = 193) Median (IQR)	*p*-Value	CKD Progression (*n* = 57) Median (IQR)	No CKD Progression (*n* = 240) Median (IQR)	*p*-Value	CKD Progression (*n* = 154) Median (IQR)	No CKD Progression (*n* = 143) Median (IQR)	*p*-Value
Serum TGF-β1 (ng/L)	21,210 (15,915–25,745)	24,200 (17,570–29,560)	0.004	20,370 (15,100–24,650)	23,740 (17,470–29,505)	0.001	20,425 (15,380–23,630)	23,320 (17,150–29,310)	0.015	22,330 (16,780–29,060)	22,080 (17,150–28,270)	0.767
Serum TGF-β2 (ng/L)	66.0 (34.8–89.2)	68.2 (36.7–100.2)	0.303	60.8 (30.6–89.3)	69.1 (38.5–94.7)	0.113	55.9 (29.9–77.8)	69.3 (37.7–94.7)	0.062	73.0 (42.4–100.5)	59.5 (30.9–88.9)	0.053
Serum TGF-β3 (ng/L)	13.9 (6.3–27.2)	14.8 (8.0–39.1)	0.506	13.0 (6.3–27.8)	14.9 (8.0–37.4)	0.512	13.5 (6.3–26.3)	14.8 (7.6–39.2)	0.421	11.8 (6.6–30.4)	16.1 (9.3–36.8)	0.286
Urine TGF-β1 (ng/L)	5.3 (2.0–14.4)	6.9 (2.8–29.7)	0.357	4.9 (1.6–14.7)	6.9 (2.9–25.1)	0.231	5.4 (1.9–29.8)	6.8 (2.8–21.1)	0.817	11.1 (3.9–29.7)	6.2 (1.6–14.8)	0.054
Urine TGF-β2 (ng/L)	11.6 (4.6–17.3)	7.8 (3.8–16.1)	0.585	9.4 (4.1–13.9)	8.3 (4.0–21.0)	0.545	10.7 (5.0–15.4)	8.3 (4.0–17.1)	0.979	10.4 (3.3–21.6)	6.8 (4.4–13.1)	0.255
Urine TGF-β3 (ng/L)	17.5 (5.4–76.2)	2.8 (1.8–15.3)	0.017	7.9 (3.1–35.8)	5.9 (1.8–42.3)	0.663	10.4 (6.9–45.0)	5.9 (1.8–34.4)	0.184	4.9 (1.8–32.2)	10.4 (2.9–42.3)	0.402

CKD, chronic kidney disease; eGFR, estimated glomerular filtration rate; uPCR, urine protein creatinine ratio; IQR, interquartile range; TGF-β, transforming growth factor beta. Data available for each biomarker as follows: serum TGF-β1 *n* = 289 (97.3%); serum TGF-β2 *n* = 245 (82.5%); serum TGF-β3 *n* = 174 (58.6%); urine TGF-β1 *n* = 89 (30.0%); urine TGF-β2 *n* = 52 (17.5%); urine TGF-β3 *n* = 53 (17.8%). Biomarker levels in remaining samples fell below the level of detection for the ELISA.

**Table 3:** The association of baseline serum and urine TGF-β with CKD progression in different models.

		eGFR Decline > 4 mL/min/1.73 m^2^/year or More				Changed to a More Advanced Stage of CKD				>30% Reduction in eGFR in 2 Years				>30% Increase in uPCR in 2 Years			
		Unadjusted		Adjusted *		Unadjusted		Adjusted *		Unadjusted		Adjusted *		Unadjusted		Adjusted *	
Baseline Biomarker	No. of Patients	OR (95% CI)	*p*-Value	OR (95% CI)	*p*-Value	OR (95% CI)	*p*-Value	OR (95% CI)	*p*-Value	OR (95% CI)	*p*-Value	OR (95% CI)	*p*-Value	OR (95% CI)	*p*-Value	OR (95% CI)	*p*-Value
Serum TGF-β1 (ng/L)	289	1.00 (1.00–1.00)	0.074	1.00 (1.00–1.00)	0.194	1.00 (1.00–1.00)	0.042	1.00 (1.00–1.00)	0.158	1.00 (1.00–1.00)	0.046	1.00 (1.00–1.00)	0.223	1.00 (1.00–1.00)	0.933	1.00 (1.00–1.00)	0.989
Serum TGF-β2 (ng/L)	245	1.00 (0.99–1.00)	0.187	1.00 (0.99–1.00)	0.165	1.00 (0.99–1.00)	0.206	1.00 (0.99–1.00)	0.181	0.99 (0.99–1.00)	0.130	0.99 (0.99–1.00)	0.178	1.01 (1.00–1.01)	0.038	1.01 (1.00–1.01)	0.052
Serum TGF-β3 (ng/L)	174	1.00 (1.00–1.00)	0.814	1.00 (1.00–1.00)	0.786	1.00 (1.00–1.00)	0.699	1.00 (1.00–1.00)	0.851	1.00 (1.00–1.00)	0.646	1.00 (1.00–1.00)	0.538	1.00 (1.00–1.00)	0.797	1.00 (1.00–1.00)	0.893
Urine TGF-β1 (ng/L)	89	0.99 (0.98–1.00)	0.174	0.99 (0.98–1.00)	0.195	0.99 (0.98–1.01)	0.312	0.99 (0.98–1.01)	0.368	1.00 (0.99–1.01)	0.611	1.00 (0.99–1.01)	0.673	1.00 (1.00–1.01)	0.412	1.00 (1.00–1.01)	0.402
Urine TGF-β2 (ng/L)	52	0.99 (0.97–1.02)	0.481	1.00 (0.97–1.03)	0.846	0.97 (0.91–1.02)	0.231	0.97 (0.92–1.03)	0.376	0.98 (0.92–1.04)	0.513	1.01 (0.95–1.08)	0.744	1.02 (0.98–1.07)	0.267	1.02 (0.98–1.06)	0.359
Urine TGF-β3 (ng/L)	53	1.01 (1.00–1.02)	0.131	1.01 (1.00–1.02)	0.046	1.00 (0.99–1.01)	0.472	1.00 (0.99–1.01)	0.662	1.00 (0.99–1.01)	0.808	1.01 (0.99–1.02)	0.315	1.00 (0.99–1.01)	0.683	1.00 (1.00–1.01)	0.416

* Adjusted for age, calcium, diuretics, calcium channel blockers and insulin. CKD, chronic kidney disease; eGFR, estimated glomerular filtration rate; uPCR, urine protein cre-atinine ratio; OR, odds ratio; CI, confidence interval; TGF-β, transforming growth factor beta.

## Data Availability

Data cannot be shared publicly because of the ethics policy at University of Witwatersrand; the participants signed a consent form, which stated that the data would be exclusively available for professional research staff. Data are available to qualifying organizations and/or individuals from the chairperson of the Human Research Ethics Committee (Medical) of the University of the Witwatersrand, Johannesburg (“Committee”) for researchers who meet the relevant ethics criteria for access to these data.

## Supplementary Materials

The following supporting information can be downloaded at https://www.mdpi.com/article/10.3390/biomedicines14010236/s1: Table S1: Baseline demographic and clinical characteristics of the study patients recruited at early stage (stage 1 and 2) by CKD progression; Table S2: Baseline demographic and clinical characteristics of the study patients recruited at later stages (stage 3a, 3b and 4) by CKD progression; Table S3: Baseline serum and urine transforming growth factor-beta of the study patients recruited at early stage (stages 1 and 2) by CKD progression using routinely used biomarkers; Table S4: Baseline serum and urine transforming growth factor-beta of the study patients recruited at later stage (stages 3a, 3b and 4) by CKD progression using routinely used biomarkers.

## Abbreviations

The following abbreviations are used in this manuscript:

ACEIs	Angiotensin Converting Enzyme inhibitors
ARBs	Aldosterone Receptors Blockers
CKD	Chronic Kidney Diseases
CMJAH	Charlotte Maxeke Johannesburg Academic Hospital
CKD-EPI	Chronic Kidney Disease Epidemiology Collaboration
ESKD	End-Stage Kidney Disease
T2DM	Type 2 Diabetes Mellitus
TGF-β	Transforming Growth Factor-β
eGFR	Estimated Glomerular Filtration Rate
IDMS	Isotope Dilution Mass Spectroscopy
uPCR	Urine Protein Creatinine Ratio
CCBs	Calcium Channel Blockers
KDIGO	Kidney Disease Improving Global Outcomes
NCDs	Non-Communicable Diseases
