# Supplementary material for: Associations of Transforming Growth Factor-β (TGF-β) with Chronic Kidney Disease Progression in Patients Attending a Tertiary Hospital in Johannesburg, South Africa
Source: Biomedicines. 2026 Jan 21;14(1):236. doi: 10.3390/biomedicines14010236 (PMC12838621; doi:10.3390/biomedicines14010236)
Supplement: Supplementary file 1 [file biomedicines-14-00236-s001.zip › biomedicines-3866048-supplementary.pdf]

Table S1. Baseline demographic and clinical characteristics of the study patients recruited at early stage (stage 1 and 2) by CKD progression.

Characteristic	CKD progression (n = 26)	No CKD progression (n = 39)	P-value
	Proportion (%) or Median (IQR)	Proportion (%) or Median (IQR)	
**Demographics:**			
Age (years)	52.5 (38 - 60)	43 (37 -59)	0.597
**Sex**			
Male	13 (50.0%)	15 (38.5 %)	
Female	13 (50.0%)	24 (61.5 %)	0.357
**Marital status**			
Single	10 (38.5 %)	16 (41.0 %)	
Married	13 (50.0 %)	20 (51.3 %)	
Widow/Widower	1 (3.9 %)	3 (7.7 %)	
Separated/Divorced	2 (7.7 %)	0	0.333
**Highest level of education**	5 (19.2 %)	0	
No formal education	6 (23.1 %)	11 (28.2 %)	
Primary	5 (19.2 %)	14 (35.9 %)	
Secondary	10 (38.5 %)	14 (35.9 %)	**0.027**
Tertiary			
**Occupation**			
Unemployed	2 (7.7 %)	8 (20.5 %)	
Domestic workers	7 (26.9 %)	7 (18.0 %)	
Self employed	6 (23.1 %)	9 (23.1 %)	
Public / Private servant	11 (42.3 %)	12 (30.8 %)	
Retired	0	3 (7.7 %)	0.304
**Clinical Variables:**			
BMI (kg/m^2)	29.7 (26.7 -33.5)	29.4 (23.3 – 33.6)	0.453
SBP (mmHg)	135.5 (126 -140)	140 (122 – 140)	0.949
DBP (mmHg)	82 (78 -90)	83 (73 – 90)	0.924
Creatinine (umol/L)	91.5 (80 -109)	85 (78 -100)	0.263
eGFR (ml/min/1.72m^2)	74 (67 -81)	75 (69 – 85)	0.533
uPCR (g/mmol)	0.012 (0.008-0.027)	0.014 (0.007 – 0.019)	0.768
FBG (mmol/L)	4.4 (4.2 – 4.7)	4.4 (4.1 – 4.9)	0.961
HbA1c (%)	7.0 (7.0 – 7.0)	7.0 (6.8 – 7.0)	0.676
Haemoglobin (g/dl)	13.7 (12.5 – 15.9)	14.3 (12.3 – 16.0)	0.525
WBC (x 10^9 cells/L)	5.49 (4.25 – 7.15)	6.01 (4.81 – 7.50)	0.139
Platelets (x 10^9 cells/L)	218.5 (200 -300)	276 (218 – 335)	**0.036**
Uric acid (mmol/L)	0.34 (0.30 – 0.42)	0.31 (0.22 – 0.35)	**0.009**
HDL cholesterol (mmol /L)	1.22 (1.02 -1.52)	1.26 (1.06 – 1.55)	0.784
Calcium (mmol /L)	2.33 (2.28 – 2.37)	2.36 (2.26 – 2.47)	0.469
Phosphate (mmol /L)	0.99 (0.89 – 1.16)	1.04 (0.94 – 1.13)	0.529
Sodium (mmol/L)	142 (139 – 144)	140 (138 – 142)	0.118
Potassium (mmol/L)	4.1 (4.0 – 4.4)	4.2 (4.0 – 4.4)	0.471
Bicarbonate (mmol/L)	24 (21 – 25)	22 (20 - 24)	**0.018**

Calcium phosphate product (mmol²/L²)	2.30 (2.10 – 2.70)	2.45 (2.18 – 2.77)	0.278
**Medications:**			
Diuretics	10 (38.5%)	12 (30.8%)	0.521
ACEIs / ARBs	8 (30.8%)	6 (15.4%)	0.139
Aldactone	0	2 (5.1%)	0.241
Calcium channel blockers	20 (76.9%)	24 (61.5%)	0.194
Statins	8 (30.8%)	14 (35.9%)	0.669
Oral Hypoglycemics	2 (7.7%)	6 (15.4%)	0.355
Insulin	4 (15.4%)	6 (15.4%)	1.00
Allopurinol	0	3 (7.7%)	0.148
Junior ASA	3 (11.5%)	5 (12.8%)	0.878
Beta blockers	9 (34.6%)	13 (33.3%)	0.915
Aldomet	5 (19.2%)	8 (20.5%)	0.899
Hydralazine	1 (3.9%)	3 (7.7%)	0.527
Nitrates (ISMN/ISDN)	0	1 (2.6%)	0.411
Doxazosin	10 (38.5%)	11 (28.2%)	0.386
Others	9 (34.6%)	10 (25.6%)	0.436

ACEIs, angiotensin converting enzyme inhibitors; ARBs, Aldosterone receptors blockers; ASA, acetylsalicylic acid; DBP, diastolic blood pressure; eGFR, estimated glomerular filtration rate; FBG, fasting blood sugar; HbA1c, glycosylated haemoglobin A1C; HDL, high density lipoprotein; IQR, interquartile range; ISMN; isosorbide mononitrate; ISDN, isosorbide dinitrate; uPCR, urine protein creatinine ratio; SBP, systolic blood pressure; WBC, white blood cells.

Table S2. Baseline demographic and clinical characteristics of the study patients recruited at later stages (stage 3a, 3b and 4) by CKD progression.

Characteristic	CKD progression (n = 116)	No CKD progression (n = 116)	P-value
	Proportion (%) or Median (IQR)	Proportion (%) or Median (IQR)	
**Demographics:**			
Age (years)	59.5 (49 – 68.5)	59.5 (49 -68)	0.515
**Sex**<br>Male<br>Female	 65 (56.0%)<br>51 (44.0%)	 63 (54.3 %)<br>53 (45.7 %)	 <br>0.792
**Marital status**<br>Single<br>Married<br>Widow/Widower<br>Separated/Divorced	 28 (24.1 %)<br>64 (55.2 %)<br>17 (14.7 %)<br>7 (6.0 %)	 35 (30.2 %)<br>57 (49.1 %)<br>16 (13.8 %)<br>8 (6.9 %)	 <br><br><br>0.734
**Highest level of education**<br>No formal education<br>Primary<br>Secondary<br>Tertiary	 13 (11.2 %)<br>24 (20.7 %)<br>46 (39.7 %)<br>33 (28.5 %)	 16 (13.8 %)<br>26 (22.4 %)<br>30 (25.9 %)<br>44 (37.9 %)	 <br><br><br>0.149
**Occupation**<br>Unemployed	 20 (17.2 %)	 14 (12.1 %)	

Domestic workers<br>Self employed<br>Public / Private servant<br>Retired	18 (15.5 %)<br>26 (22.4 %)<br>40 (34.5 %)<br>12 (10.3 %)	24 (20.7 %)<br>24 (20.7 %)<br>44 (37.9 %)<br>10 (8.6 %)	0.668
**Clinical Variables:**			
BMI (kg/m²)	30.6 (26.8 -35.8)	29.8 (26.5 – 33.2)	0.483
SBP (mmHg)<br>DBP (mmHg)	140 (133.5 -140)<br>83 (74 -90)	140 (127 – 140)<br>82 (72 – 90)	**0.044**<br>0.287
Creatinine (umol/L)	156 (133 -187.5)	147 (122 -174)	**0.021**
eGFR (ml/min/1.72m²)	35 (31 -43.5)	39.5 (32 – 47.5)	**0.011**
uPCR (g/mmol)	0.051 (0.021-0.095)	0.018 (0.01 – 0.038)	**<0.001**
FBG (mmol/L)	4.5 (4.2 – 5.6)	4.4 (4.2 – 4.9)	0.373
HbA1c (%)	7.0 (6.8 – 7.0)	7.0 (6.6 – 7.0)	0.383
Haemoglobin (g/dl)	12.8 (11.5 – 14.1)	13.6 (12.2 – 14.8)	**0.018**
WBC (x 10^9 cells/L)	6.16 (5.20 – 7.91)	6.48 (4.81 – 7.85)	0.683
Platelets (x 10^9 cells/L)	256.5 (222 -327.5)	263.5 (216 – 319)	0.877
Uric acid (mmol/L)	0.45 (0.38 – 0.53)	0.43 (0.36 – 0.51)	0.357
HDL cholesterol (mmol /L)	1.15 (0.98 -1.43)	1.24 (1.00 – 1.59)	0.163
Calcium (mmol /L)	2.29 (2.20 – 2.39)	2.34 (2.25 – 2.42)	**0.007**
Phosphate (mmol /L)	1.13 (0.94 – 1.30)	1.01 (0.88 – 1.17)	**0.002**
Sodium (mmol/L)	140 (138.5 – 143)	141 (138 – 143)	0.666
Potassium (mmol/L)	4.4 (4.1 – 4.7)	4.2 (3.75 – 4.65)	**0.041**
Bicarbonate (mmol/L)	22 (20 – 24)	22 (20 - 24)	0.506
Calcium phosphate product (mmol²/L²)	2.56 (2.16 – 2.92)	2.38 (1.98 – 2.70)	**0.024**
**Medications:**			
Diuretics	80 (69.0%)	51 (44.0%)	**<0.001**
ACEIs / ARBs	25 (21.6%)	21 (18.1%)	0.510
Aldactone	6 (5.2%)	3 (2.6%)	0.308
Calcium channel blockers	101 (87.1%)	93 (80.2%)	0.156
Statins	73 (62.9%)	56 (48.3%)	**0.025**
Oral Hypoglycemics	12 (10.3%)	16 (13.8%)	0.420
Insulin	41 (35.3%)	17 (14.7%)	**<0.001**
Allopurinol	15 (12.9%)	16 (13.8%)	0.847
Junior ASA	38 (32.8%)	23 (19.8%)	**0.025**
Beta blockers	70 (60.3%)	50 (43.1%)	**0.009**
Aldomet	30 (25.9%)	26 (22.4%)	0.539
Hydralazine	10 (8.6%)	5 (4.3%)	0.182
Nitrates (ISMN/ISDN)	4 (3.5%)	2 (1.7%)	0.408
Doxazosin	54 (46.6%)	59 (50.9%)	0.511
Others	36 (31.0%)	44 (37.9%)	0.269

ACEIs, angiotensin converting enzyme inhibitors; ARBs, Aldosterone receptors blockers; ASA, acetylsalicylic acid; DBP, diastolic blood pressure; eGFR, estimated glomerular filtration rate; FBG, fasting blood sugar; HbA1c, glycosylated haemoglobin A1C; HDL, high density lipoprotein; IQR, interquartile

range; ISMN; isosorbide mononitrate; ISDN, isosorbide dinitrate; uPCR, urine protein creatinine ratio; SBP, systolic blood pressure; WBC, white blood cells.

Table S3. Baseline serum and urine transforming growth factor-beta of the study patients recruited at early stage (stages 1 and 2) by CKD progression using routinely used biomarkers.

	eGFR decline > 4 ml/min/1.73 m2/year or more			Changed to a more advanced stage of CKD			> 30% reduction in eGFR in 2 years			> 30% increase in uPCR in 2 years		
**Baseline Characteristics**	**CKD progression (n = 26)**	**No CKD progression (n = 39)**	**p-value**	**CKD progression (n = 15)**	**No CKD progression (n = 50)**	**p-value**	**CKD progression (n = 6)**	**No CKD progression (n = 59)**	**p-value**	**CKD progression (n = 28)**	**No CKD progression (n = 37)**	**p-value**
	Median (IQR)	Median (IQR)		Median (IQR)	Median (IQR)		Median (IQR)	Median (IQR)		Median (IQR)	Median (IQR)	
Serum TGF-β1 (ng/L)	23380 (20370 – 30190)	27620 (21580 – 30660)	0.256	23560 (21290 – 33030)	26090 (21240 – 30570)	0.943	28295 (21760 – 42560)	25960 (21240 – 30370)	0.333	28520 (23225 – 30515)	23970 (19055 – 31180)	0.140
Serum TGF-β2 (ng/L)	66.3 (34.8 – 87.1)	79.1 (44.7 – 119.3)	0.193	41.2 (34.8 – 75.3)	82.8 (47.1 – 106.9)	0.106	58.2 (38.0 – 77.8)	73.7 (34.8 – 103.5)	0.677	86.6 (66.7 – 106.9)	54.5 (27.1 – 92.7)	0.080
Serum TGF-β3 (ng/L)	10.4 (2.3 – 20.6)	16.0 (4.9 – 35.4)	0.260	3.7 (1.2 – 13.0)	14.8 (5.2 – 35.4)	0.066	7.1 (1.2 – 88.4)	12.4 (4.0 – 28.9)	0.616	11.5 (4.0 – 18.0)	14.9 (3.7 – 47.7)	0.301
Urine TGF-β1(ng/L)	11.6 (1.8 – 21.4)	11.5 (2.3 – 122.4)	0.505	8.9 (1.3 – 21.4)	11.5 (2.3 – 71.8)	0.390	6.3 (1.3 – 26.1)	11.6 (2.3 – 58.0)	0.558	17.4 (11.5 – 122.4)	6.8 (0.9 – 26.1)	0.091
Urine TGF-β2 (ng/L)	4.7 (4.6 – 12.6)	10.9 (3.8 – 34.3)	0.517	4.7 (4.6 – 4.7)	12.6 (3.8 – 34.3)	0.352	–[b]	9.2 (3.8 – 34.3)		27.8 (7.1 – 69.3)	5.9 (3.8 - 12.6)	0.160
Urine TGF-β3 (ng/L)	138.0 (0.8 – 275.1)	4.4 (0.8 – 13.6)	0.686	0.8 (0.8 – 0.8)	7.9 (0.8 – 1.3)	0.281	–[b]	4.4 (0.8 – 15.3)		7.9 (0.8 – 15.3)	0.9 (0.8 – 11.8)	0.747

[a] Data available for each biomarker as follows: Serum TGF-β1 n=64 (98.5%); Serum TGF-β2 n=57(87.7%); Serum TGF-β3 n=43 (66.2%); Urine TGF-β1 n=25 (38.5%); Urine TGF-β2 n=19 (29.2%); Urine TGF-β3 n=10 (15.4%). Biomarker levels in remaining samples fell below the level of detection for the ELISA. [b] No individuals with data in this category.

Table S4. Baseline serum and urine transforming growth factor-beta of the study patients recruited at later stage (stages 3a, 3b and 4) by CKD progression using routinely used biomarkers.

	**eGFR decline > 4 ml/min/1.73 m2/year or more**			**Changed to a more advanced stage of CKD**			**> 30% reduction in eGFR in 2 years**			**> 30% increase in uPCR in 2 years**		
**Baseline Characteristics**	**CKD progression (n = 116)**	**No CKD progression (n = 116)**	**p-value**	**CKD progression (n = 89)**	**No CKD progression (n = 143)**	**p-value**	**CKD progression (n = 51)**	**No CKD progression (n = 181)**	**p-value**	**CKD progression (n = 126)**	**No CKD progression (n = 106)**	**p-value**
	**Median (IQR)**	**Median (IQR)**		**Median (IQR)**	**Median (IQR)**		**Median (IQR)**	**Median (IQR)**		**Median (IQR)**	**Median (IQR)**	
Serum TGF-β1 (ng/L)	20220 (14770 – 24850)	23255 (16360 – 29280)	**0.026**	19035 (14700 – 23700)	22840 (16410 – 29280)	**0.004**	19435 (14700 – 22600)	22080 (15840 – 29130)	**0.023**	20775 (15440 – 26780)	21840 (15840 – 28080)	0.426
Serum TGF-β2 (ng/L)	66.0 (33.7 – 92.6)	67.0 (35.0 – 92.8)	0.656	62.2 (30.4 – 92.1)	67.0 (37.7 – 93.3)	0.364	55.9 (29.9 – 76.3)	68.8 (37.7 – 94.7)	0.070	68.3 (39.9 – 99.1)	60.3 (31.7 – 87.5)	0.230
Serum TGF-β3 (ng/L)	16.1 (8.0 – 28.3)	14.3 (9.0 – 43.0)	0.852	15.5 (8.0 – 31.3)	15.5 (8.7 – 37.4)	0.933	15.0 (7.4 – 26.3)	15.5 (9.0 – 43.3)	0.335	12.4 (7.4 – 43.0)	16.1 (10.5 – 28.3)	0.396
Urine TGF-β1(ng/L)	4.9 (2.3 – 14.2)	6.7 (2.9 – 19.7)	0.496	4.9 (1.6 – 14.4)	6.5 (2.9 – 17.3)	0.425	4.9 (2.3 – 33.5)	5.6 (2.9 – 14.2)	0.831	5.6 (3.8 – 23.1)	5.6 (1.6 – 12.8)	0.160
Urine TGF-β2 (ng/L)	11.8 (6.5 – 17.3)	7.3 (4.0 – 8.9)	0.109	10.7 (3.5 – 14.4)	7.5 (4.2 – 16.1)	0.870	10.7 (5.0 – 15.4)	7.6 (4.2 – 13.5)	0.769	8.9 (3.3 – 16.9)	7.1 (5.9 – 13.5)	0.613
Urine TGF-β3 (ng/L)	17.5 (5.9 – 72.4)	2.9 (1.8 – 16.4)	**0.022**	8.9 (3.4 – 45.0)	5.9 (1.8 – 45.1)	0.499	10.4 (6.9 – 45.0)	5.9 (1.8 – 42.3)	0.232	4.9 (1.8 – 47.9)	15.5 (4.3 – 43.7)	0.192

[a] Data available for each biomarker as follows: Serum TGF-β1 n=225 (97.0%); Serum TGF-β2 n=188 (81.0%); Serum TGF-β3 n=131 (56.5%); Urine TGF-β1 n=64 (27.6%); Urine TGF-β2 n=33 (14.2%); Urine TGF-β3 n=43 (18.5%). Biomarker levels in remining samples fell below the level of detection for the ELISA
